# Intrapericardial Extralobar Pulmonary Sequestration: A Case Report and Systematic Review of a Unique Embryologic Variant

**DOI:** 10.3390/jcm15030932

**Published:** 2026-01-23

**Authors:** Margherita Roveri, Giada Pedroni, Alessandra Preziosi, Luigi Arcieri, Stefano Marianeschi, Francesco Macchini, Andrea Zanini

**Affiliations:** 1 Pediatric Surgery, ASST Grande Ospedale Metropolitano Niguarda, 20162 Milan, Italy; 2 Pediatric Surgery Specialty School, University of Milan, 20162 Milan, Italy; 3 Pediatric Cardiac Surgery, ASST Grande Ospedale Metropolitano Niguarda, 20162 Milan, Italy

**Keywords:** intrapericardial extralobar pulmonary sequestration, intrapericardial lesion, congenital teratoma, congenital lung malformations, hybrid lesion, pediatric thoracic surgery

## Abstract

**Background**: Intrapericardial extralobar pulmonary sequestration (ELPS) is an exceptionally rare congenital malformation. The location may mimic neoplastic lesions and poses diagnostic and surgical challenges. We present a new case and a systematic review of the literature. **Case Presentation**: A 3-month-old male infant was referred for evaluation of a congenital intrathoracic mass suspected to be an extralobar sequestration. However, intrapericardial location was not recognized. MRI and CT demonstrated a circumscribed lesion with arterial supply from the right pulmonary artery. Thoracoscopic exploration was attempted but converted to sternotomy. The mass was excised en bloc. Histopathological analysis confirmed extralobar pulmonary sequestration with cystic components, consistent with a hybrid lesion. Postoperative recovery was uneventful. **Methods**: A systematic literature review was conducted according to PRISMA guidelines across PubMed, Scopus and Embase databases, including only histologically confirmed intrapericardial ELPS. **Results**: Ten cases were identified. Including the present case, eleven cases have been reported. Prenatal detection occurred in 54% of cases. Fetal demise occurred in two cases due to cardiac tamponade. Aberrant arterial supply originated from the pulmonary arteries in 54% of patients and venous drainage into the right atrium or superior vena cava in 45%. Surgery via sternotomy was performed in all cases with excellent outcomes. **Conclusions**: Intrapericardial ELPS is an exceptionally rare but surgically curable entity. Early recognition and complete resection are essential to prevent life-threatening complications. This systematic review highlights a consistent vascular pattern supporting its classification as a unique embryologic variant within the CPAM–sequestration spectrum.

## 1. Introduction

Intrapericardial masses are rare in the pediatric population and represent a heterogeneous group of lesions. Neoplastic forms include teratomas, rhabdomyomas, and thymomas, whereas congenital lung malformations (CLMs) such as pulmonary airway malformations (CPAM), pulmonary sequestrations (PS), and bronchogenic cysts are particularly uncommon within the pericardial space [1]. Vascular and lymphatic malformations have also been described, although they remain exceptional findings [2].

Most intrapericardial masses are diagnosed prenatally, typically during the second trimester. However, establishing a precise prenatal differential diagnosis can be challenging, and in the postnatal period the diagnosis often remains elusive, frequently requiring advanced imaging modalities and a multidisciplinary evaluation to ensure accurate characterization and optimal management [3].

Extralobar pulmonary sequestrations (ELPS) typically develop within the thoracic cavity but they may arise in atypical or ectopic sites. Intrapericardial location, however, has only been seldomly reported [4].

We present a case of congenital intrapericardial mass confirmed as ELPS and we provide a review the literature to outline diagnostic and management considerations, as well as to offer insights and suggestions for this unique variant.

## 2. Materials and Methods

A comprehensive literature review was conducted according to the Preferred Reporting Items for Systematic Reviews and Meta-Analyses (PRISMA) guidelines, with the objective of identifying all previously reported cases of histologically confirmed intrapericardial extralobar pulmonary sequestration. Three electronic databases were searched: PubMed, Embase, and Scopus. The search was performed without restrictions on publication date or patient age and was updated to October 2025. This systematic review was conducted in accordance with PRISMA guidelines (Supplemental File S1) [5].

The following keyword combinations and Boolean operators were used: “intrapericardial pulmonary sequestration”, “intrapericardial extralobar sequestration”, “ectopic pulmonary sequestration”, “intrapericardial bronchopulmonary sequestration”, and “pericardial pulmonary sequestration”. Reference lists of relevant articles were also manually screened to identify additional eligible cases.

Inclusion criteria were: articles published in English; original reports of intrapericardial extralobar pulmonary sequestration confirmed by histopathological examination; and availability of individual patient-level data on clinical presentation, imaging findings, anatomical vascular supply, surgical management, and outcomes. Exclusion criteria included duplicate publications, review articles without original cases, pericardiac but extra pericardial lesions, intralobar sequestrations, and congenital lung malformations without sequestration features.

When both an individual case report and a subsequent case series from the same institution were available, only the most comprehensive report was included in the final analysis, while earlier reports were reviewed to extract any additional clinical information not present in the later publication. Data from all included studies were independently reviewed and extracted into a dedicated database for qualitative synthesis and comparison with the present case.

## 3. Case Presentation

A 3-month-old male infant was referred to our tertiary care center following the antenatal detection of a congenital thoracic mass. Fetal ultrasound and magnetic resonance imaging (MRI) demonstrated a well-circumscribed, heterogeneous lesion located in the right upper chest with focal cystic areas, suggestive of an extralobar pulmonary sequestration. However, the exact anatomical relationship of the mass to the pericardium could not be determined prenatally, and an intrapericardial location was not suspected at the presenting Institution. The prenatal diagnostic work-up was performed at another institution, and detailed information regarding the reasons why an intrapericardial location was not considered is not available.

The pregnancy progressed uneventfully, and the infant was delivered at 39 weeks of gestation with a birth weight of 3150 g and Apgar scores of 9 and 10 at 1 and 5 min, respectively. Clinical examination at birth was unremarkable, with normal heart sounds, clear lung fields, and no respiratory distress. Initial postnatal imaging performed at the referring institution confirmed the presence of a right upper chest lesion: pulmonary sequestration and hybrid congenital lung malformation were the leading hypothesis.

Following admission to our center, a transthoracic echocardiogram unexpectedly revealed that the lesion was located entirely within the pericardial sac, adherent to the right atrium and superior vena cava. This unexpected finding significantly altered the diagnostic approach. The differential diagnosis at this stage included intrapericardial vascular malformation, pericardial teratoma, and cardiac rhabdomyoma. Pulmonary sequestration or a hybrid congenital lung lesion were considered extremely unlikely due to their exceptionally rare intrapericardial presentation. Serum alpha-fetoprotein (AFP) levels and bHCG were within the expected physiological range for age and displayed a progressive decline.

**Figure 1:**
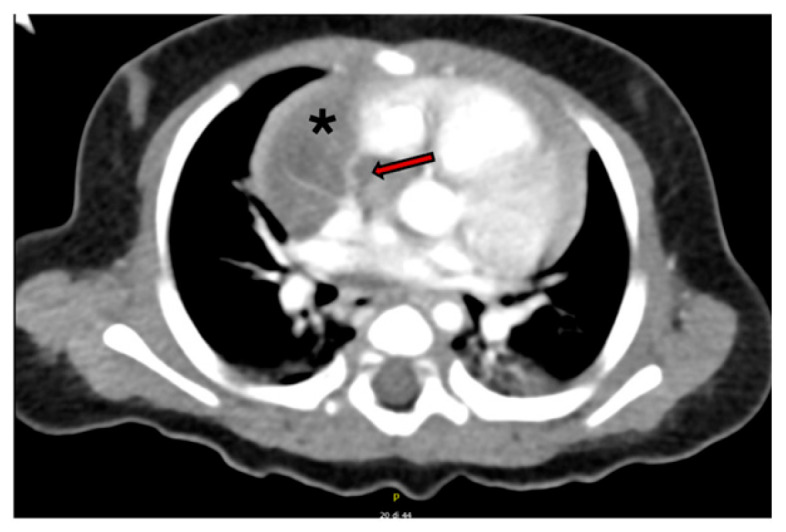
Angio- CT scan. Right paracardiac lesion with well-defined and regular margins, tightly adherent to the mediastinal structures. It indents the lateral wall of the right atrium and compresses the superior vena cava. Its average density is supraliquid in nature; however, within the lesion the presence of some vascular structures originating from the right pulmonary artery is confirmed. Red arrow: vascular pedicle, Asterisk: lesion.

**Figure 2:**
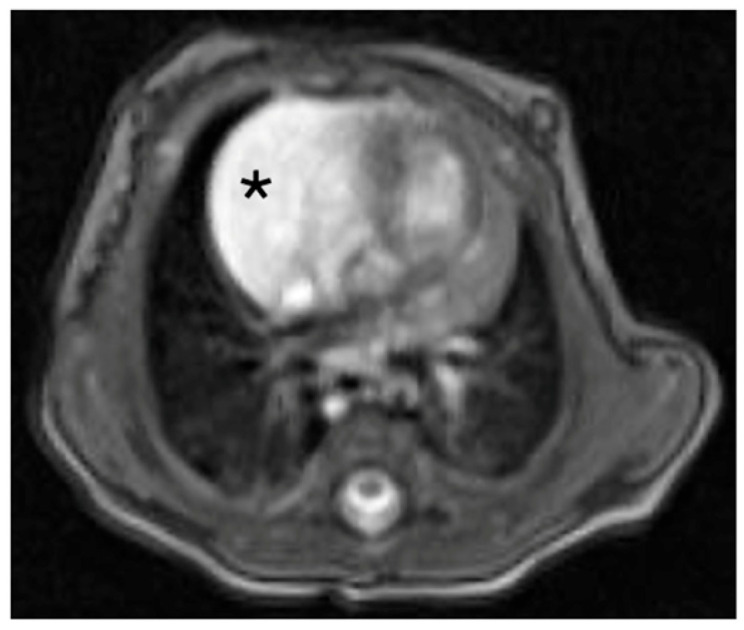
MRI T2-weighted. Right paracardiac mass with well-defined margins, showing hyperintense signal on T2-weighted images and containing some branching inhomogeneities suggestive of a possible vascular nature. Asterisk: lesion.

A contrast-enhanced computed tomography (CT) scan confirmed the presence of a well-circumscribed intrapericardial mass measuring 37 × 33 × 23 mm, closely abutting the ascending aorta, right atrium, and superior vena cava, without evidence of invasion of adjacent structures. A thin feeding artery arising from the right pulmonary artery was identified (Figure 1 and Figure 2).

Given the benign radiological features, the slow interval growth of the lesion, the normal tumor markers, and the apparent feasibility of complete resection, the multidisciplinary team opted for surgical exploration with the aim of an upfront surgical excision.

**Figure 3:**
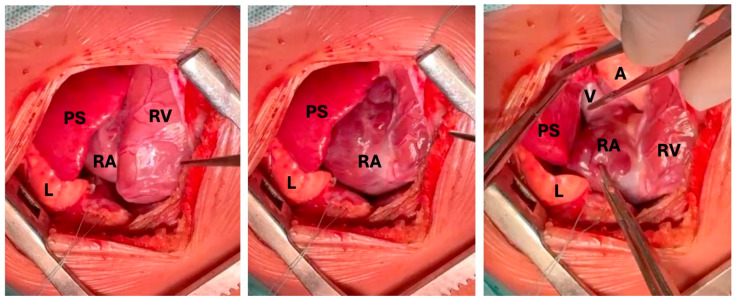
Intraoperative view of the lesion. The lesion is clearly distinct from the normal lung parenchyma. The sequestration is completely separated from the cardiac surface, with the vascular pedicle visible. RV: right ventricle, RA: right atrium, L: lung, PS: pulmonary sequestration, V: vascular pedicle, A: aorta.

At four months of age, the patient underwent surgery. The procedure was initiated thoracoscopically: the intrapericardial location of the mass was confirmed. After pericardiotomy, the lesion appeared predominantly cystic, but no clear dissection planes were visible from the right atrium. Due to the deemed high risk of resection and rupture, conversion to a median sternotomy was performed. Upon open exposure, the mass was found lying over the right atrium and superior vena cava but without direct invasion or adherence to cardiac structures or great vessels. A single vascular pedicle originating from the right pulmonary artery was identified, ligated, and divided, allowing a complete excision of the lesion without intraoperative complications (Figure 3).

The postoperative course was uneventful. The infant was extubated in the operating room, did not require intensive care admission, and was discharged home on postoperative day three.

Histopathological examination confirmed the diagnosis of extralobar pulmonary sequestration (EPS) with both solid and cystic components filled with mucoid fluid, consistent with a hybrid lesion. At six-month follow-up, the patient remained asymptomatic, with normal cardiac function and no evidence of recurrence.

## 4. Results

**Figure 4:**
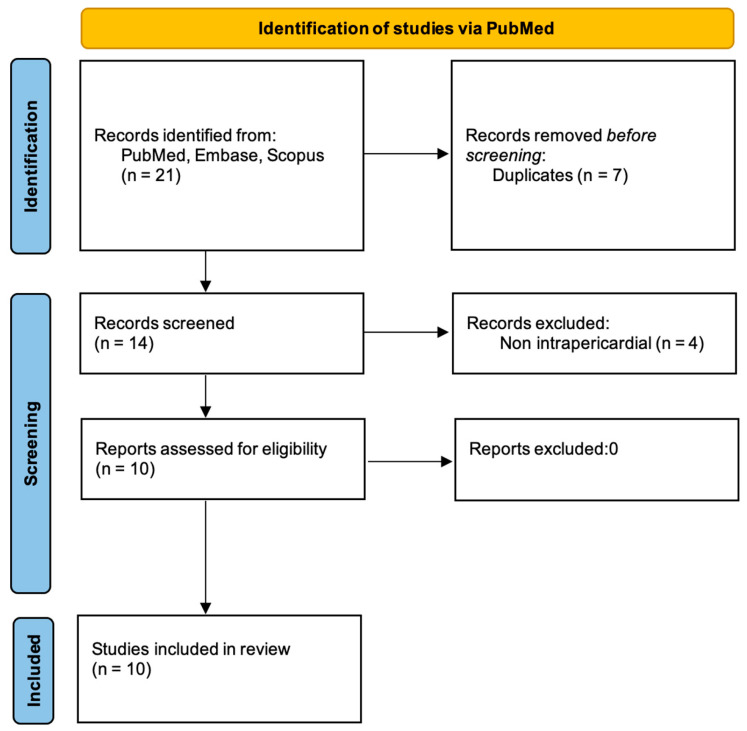
PRISMA flow diagram of study selection. Flow diagram showing the identification, screening, eligibility, and inclusion of studies on intrapericardial extralobar pulmonary sequestration, with reasons for exclusion reported at each stage.

A total of 21 records were identified across the three databases. After removal of duplicates and screening of titles and abstracts, 10 full-text articles were assessed for eligibility. All 10 studies met the inclusion criteria and were included in the final qualitative synthesis. The study selection process was documented in a PRISMA flow diagram. (Figure 4).

Our literature review identified 10 cases of intrapericardial sequestrations: 2 in adult patients [6,7], 6 in newborns [4,8,9,10,11,12] and 2 in stillborn [13,14]. Our case brings the total to 11 (Table 1).

Ten cases of intrapericardial extralobar pulmonary sequestration meeting the inclusion criteria were identified in the literature. Including the present case, a total of 11 cases has been reported (Table 1).

Six out of the eleven cases (54%) were diagnosed prenatally, at a mean gestational age of 23.5 ± 4.03 weeks. Two cases resulted in intrauterine fetal demise: 1 due to hydrops and cardiac tamponade at 29 weeks’ gestation, and 1 following elective pregnancy termination despite an asymptomatic course.

Among liveborn patients, the mean gestational age at delivery was 33 ± 5 weeks, with a mean birth weight of 2726 ± 832 g. Five neonates (45%) were asymptomatic at birth, whereas both adult patients were symptomatic, presenting with chest pain. Symptomatic neonatal presentations included respiratory distress, cyanosis, tachypnea, and bilateral pneumothorax in two cases.

Cross-sectional imaging (CT or MRI) was performed in all cases. The lesion was predominantly cystic in 7 cases (64%) and solid in 2. Pericardial effusion was reported in 4 patients (36%), including 2 detected prenatally, 1 of which resulted in fetal death.

A preoperative diagnosis of intrapericardial pulmonary sequestration was suspected in 4 cases, including ours. The remaining cases were initially misdiagnosed as pericardial teratoma, congenital pulmonary airway malformation, or bronchogenic cyst.

Among pediatric patients, the mean age at surgery was 42.8 ± 55.9 days. In all resected cases, the procedure was performed via median sternotomy, the mass did not show infiltration to the surrounding structures and complete excision was achieved without reported intraoperative complications. The mass was located on the right side of the heart in 5 cases (45%), on the left in 3 patients (27%), while in the remaining cases, the specific side was not specified.

The aberrant systemic arterial supply originated from the pulmonary arteries in 6 patients (54%), including the present case, and from branches of the thoracic aorta in 2 cases. No clear arterial supply was reported in 2 cases. Venous drainage was most commonly directed into the superior vena cava (4/11: 36%), followed by direct drainage into the left atrium in 1 case; it was not specified in the remaining reports.

An atretic bronchial connection was identified in 3 cases. The median lesion size was 45 × 30 × 23 mm. Histopathological examination confirmed extralobar pulmonary sequestration in all cases; 1 lesion was classified as a hybrid lesion with associated congenital pulmonary airway malformation type II.

## 5. Discussion

Congenital intrapericardial masses are rare and heterogeneous entities, and their proximity to the heart and great vessels poses major diagnostic and therapeutic challenges. Regardless of their malignant or benign nature, intrapericardial lesions have the potential to generate significant mass effect, compromising venous return and cardiac filling, and may lead to pericardial effusion, fetal hydrops, or cardiac tamponade [1,15].

Early recognition of these hemodynamic disturbances is crucial, as rapid cardiovascular compromise can ensue and become life-threatening [1].

Because the available evidence mainly derives from isolated case reports, systematic comparison of published cases is essential to improve diagnostic accuracy and guide management strategies. This review highlights recurring patterns that may assist clinicians in optimizing all aspects of patient care, from diagnosis to therapeutic intervention.

Among intrapericardial masses, teratoma is the most common entity. Its characteristic heterogeneous solid–cystic architecture often overlaps with other congenital lesions, explaining why teratoma is the most frequent preoperative diagnosis in published reports [16]. Differential diagnosis includes rhabdomyoma, vascular or lymphatic malformations, pericardial cysts, and ectopic thymic tissue [17]. Much more rarely, congenital pulmonary malformations such as extralobar pulmonary sequestration (ELPS), CPAM, bronchogenic cysts, or hybrid lesions have also been seldomly described in the pericardial cavity [18]. Maintaining clinical cognizance of these rare entities is essential to avoid diagnostic misclassification and suboptimal patient management.

**Figure 5:**
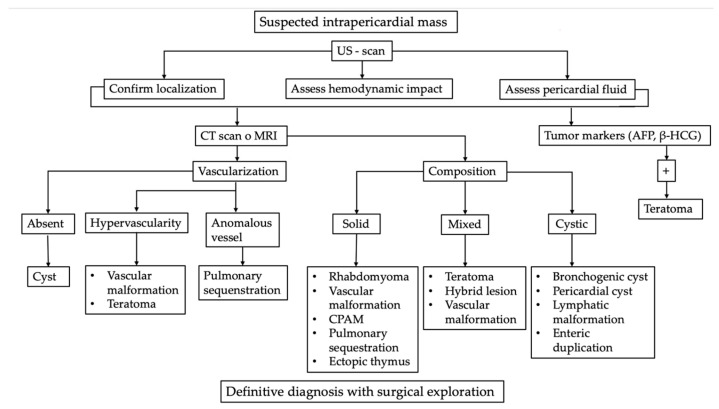
Intrapericardial masses diagnostic algorithm. First assessment with US scan followed by tumor markers testing and anatomical evaluation with CT scan or MRI.

The main differential diagnoses of intrapericardial masses in newborns and infants are summarized in the diagnostic algorithm shown in Figure 5.

Diagnostic approach to a suspected intrapericardial mass should start with Ultrasound. US-scan represents the essential first-line assessment, allowing confirmation of mass localization, detection of associated pericardial effusion, and evaluation of any hemodynamic compromise [1,19].

Second-line imaging with computed tomography (CT) or magnetic resonance imaging (MRI) is recommended to further characterize the lesion, with particular emphasis on vascular characteristics and tissue composition. Avascular lesions are typically consistent with simple cysts, whereas hypervascular masses may suggest vascular malformations or teratomas. Lesions supplied by anomalous systemic vessels are highly suggestive of pulmonary sequestration [19,20].

Assessment of mass composition further refines the differential diagnosis. Solid or cystic morphology helps direct suspicion toward specific entities, while mixed composition most commonly points to teratomas or hybrid lesions [16,20].

Tumor marker evaluation, including alpha-fetoprotein and β-human chorionic gonadotropin, may support the diagnosis of germ cell tumors, particularly teratomas. Despite advances in imaging techniques and laboratory investigations, definitive diagnosis often requires surgical exploration followed by histopathological examination [1,19].

ELPS consists of non-functioning pulmonary tissue with aberrant vascularization and absence of communication with the tracheobronchial tree [20,21]. While ELPS usually localizes between the lung and diaphragm, intrapericardial occurrence is exceptionally rare [22].

In our series, more than half of the cases of our series were diagnosed prenatally, typically in the second trimester, when routine fetal ultrasound revealed a heterogeneous mass adjacent to the heart. However, a key clinical challenge is to distinguish between intra- and extra-pericardial localization, which is often not feasible prenatally. Definitive prenatal diagnosis is rarely achievable.

Retrospective reviews of prenatal imaging have occasionally revealed aberrant vascular supply, usually originating from the pulmonary artery or systemic circulation, not Retrospective reviews of prenatal imaging have occasionally revealed aberrant vascular supply that had not been detected during the initial assessment, originating from the pulmonary artery or systemic circulation, not detected during initial evaluations. Identification of such vessels, particularly via color Doppler ultrasonography or fetal MRI, should raise suspicion for pulmonary sequestration [23,24]. Careful interrogation of vascular anatomy represents one of the most important diagnostic clues in this setting [23,24].

The clinical course and progression of intrapericardial ELPS appears highly variable. Two fetuses died in utero due to hydrops and tamponade [25,26], whereas other patients remained asymptomatic at birth or even until adulthood [6,7]. Notably, neither lesion size nor postnatal growth consistently correlated with symptom severity.

Consequently, close prenatal and postnatal surveillance is warranted even in apparently stable cases.

Postnatally, echocardiography is crucial to assess cardiac anatomy, pericardial involvement and hemodynamic consequences. CT and MRI play a key role in defining the vascular pedicle and anatomical definition [3,21,27]. Despite advanced imaging, a preoperative suspicion of intrapericardial ELPS was reported in only 36% of cases.

Surgical resection represents the treatment of choice and is universally curative. The favorable surgical outcomes reported in all patients support the rationale for early surgical excision, which provides a definitive histopathological diagnosis and prevents infections and cardiovascular complications [28,29]. Once the mass is identified and deemed resectable a proactive surgical approach should be undertaken.

In all previously reported cases, surgical resection was performed via median sternotomy. In the present case, a thoracoscopic approach was attempted, marking the first reported minimally invasive attempt for intrapericardial ELPS. Thoracoscopy offers recognized advantages, including reduced surgical trauma and faster recovery [30]. However, it is limited by restricted maneuverability, higher risk of cyst rupture, and potential compromise of oncological principles [31]. In our case the lesion appeared predominantly cystic, and due to poor visibility, no clear dissection plane could be identified from the right atrium. In our case the lesion appeared predominantly cystic, and due to poor visibility, no clear dissection plane could be identified from the right atrium. The cystic components precluded safe grasping, resulting in a high risk of rupture and potential spillage. In addition, an adequate working space could not be achieved because the patient did not tolerate single-lung ventilation.

Considering the diagnostic uncertainty and the high risk of incomplete resection or rupture, conversion to a median sternotomy was deemed the safest option. Following adequate exposure was achieved, the lesion was found to be in contact with—but not adherent—to the right atrium and the vascular pedicle was easily clamped and divided. In retrospect, we believe the resection could have been carried out thoracoscopically with improved exposure. Our experience does not support routine thoracoscopy in suspected intrapericardial ELPS; instead, it suggests that a minimally invasive attempt can be reasonable in highly selected cases, provided there is a low threshold for conversion.

Given its extreme rarity and diagnostic uncertainty, intrapericardial ELPS should initially be managed as a potentially neoplastic lesion. Until histopathological confirmation is obtained, surgical management should follow oncologic principles, including en bloc resection with intact capsule and secure vascular control. Conversion from a minimally invasive to an open approach should be regarded as an appropriate strategy to ensure safety and diagnostic accuracy [32,33].

Analysis of vascular anatomy revealed pulmonary arterial supply in more than half of the cases, including the present one, contrasting with the classical systemic arterial origin of ELPS. This suggests the possibility of a distinct embryological variant. Venous drainage most occurred into the superior vena cava or right atrium, resulting in a right-to-right shunt and potentially explaining the absence of cardiac overload in most patients. The review of the macroscopic and microscopic hystological examination revealed cystic components or bronchial remnants in 64% of lesions, supporting the hypothesis that intrapericardial sequestration may represent part of a broader spectrum of foregut-derived malformations, rather than a completely distinct entity [34,35].

Although hybrid lesions combining ELPS and CPAM components have been reported both in intrapericardial and extrapericardial locations, current evidence does not support a role of the intrapericardial environment in driving hybrid lesion development. The presence of CPAM-like elements is more likely related to early abnormalities of foregut and lung bud development rather than to secondary anatomical positioning. Given the rarity of intrapericardial ELPS and the limited number of described cases, no causal relationship between intrapericardial location and hybrid histology can currently be established [36,37].

Recognition of this rare entity may allow a more accurate prenatal counseling, more appropriate surgical planning, and avoidance of potentially hazardous intraoperative strategies.

This study represents the first review exclusively focused on histologically confirmed intrapericardial ELPS and highlights recurring anatomical and embryological patterns. The main limitations include the small number of reported cases, retrospective data collection, and incomplete reporting, which preclude statistical analysis and underscore the need for standardized reporting of future cases.

## 6. Conclusions

Intrapericardial extralobar pulmonary sequestration, although extremely rare, should be considered in the differential diagnosis of congenital intrapericardial masses. Preoperative distinction from neoplastic lesions is challenging; therefore, surgical management should follow oncologic principles to ensure a safe and complete resection. This case, increasing the total number of reported patients to eleven, suggests a recurring pattern of pulmonary arterial supply and right-sided venous drainage. Moreover, it supports the notion that intrapericardial ELPS may belong to the broader spectrum of hybrid CPAM–sequestration malformations, highlighting the importance of careful histopathological evaluation.

## Figures and Tables

**Table 1:** Summary of Review’s findings. “nd”: non-defined, “GA”: gestional age, “IUD”: intrauterine death.

Reference	GA	Birth Weight (g)	Sex	Prenatal Diagnosis	Symptoms	Characteristic of the Mass	Preoperative Diagnosis	Age at Surgery	Surgical Approach	Side of the Mass	Size of Mass (mm)	Arterial Supply	Venous Drainage	Airways Connections or Stalks	Hystopath
1, Levi [8]	42	4050	M	no	dyspena, cyanosis	solid mass	intrapericardial mass hamartoma	2 days	open thoracotomy and midsternotomy	left	45 × 58 × 15	no connection	no connection		pulmonary sequestration
2, Ahn CM [7]	nd	nd	M	no	right chest pain and weakness	multicystic mass	mediastinal mass	27 years	open	right	80 × 80	subclavian artery	not mentioned		pulmonary sequestration
3, Hayashi AH [9]	(36–40)	4250	M	no	dyspnea, bilateral pneumothoraces	cystic mass	mediastinal mass	21 days	open sternotomy	right	30 × 30 × 40	no connection	no connection	Attached to the distal trachea by a fibrocartilagenous stalk	pulmonary sequestration
4, Wax JR [10]	39	3400	F	yes (29 week)	no	solid and cystic mass	teratoma/ sequestration	5 days	open sternotomy	left	31 × 24 × 23	left pulmonary artery	not mentioned		pulmonary sequestration
5, Yildiz K [13]	29 (IUD)	-	-	yes (29 week)	fetal hydrops	solid mass	pericardial teratoma	none	no	right	40 × 30 × 30	not mentioned	not mentioned		pulmonary sequestration
6, Al-Mudaffer M [11]	39	3500	M	no	bilateral pneumothoraces, tachycardia, apnea	solid and cystic mass	intrapericardial mass	11 days	open sternotomy	nd	69 × 57 × 18	innominate artery	supra vena cava		ELPS with associated congenital pulmonary adenomatoid malformation type II (hybrid lesion)
7, De Vreede I11	39	2770	M	yes (20 weeks)	no	solid and cystic mass	CCAM or sequestration	21 days	open sternotomy	nd	70 × 20	right pulmonary artery	vena cava	Connection with the trachea through an atretic side branch of the trachea	pulmonary sequestration
8, Yanagisawa [4]	38	2916	M	yes (23 weeks)	no	solid and cystic mass	intrapericardial sequestration/ teratoma / bronchogenic cyst	90 days	open sternotomy	left	45 × 30 × 20	right pulmonary artery	supra vena cava		pulmonary sequestration
9, Wei Y [6]	nd	nd	F	no	Dyspnea, chest pain for 6 m	cystic mass	pericardial cyst	24 years	open sternotomy	right	180 × 120 × 50	Bronchial artery and pulmonary artery	left atrium	3-mm bronchium from the mass connecting to the trachea	pulmonary sequestration
10, Yan YN [14]	25 (abortion)	-	F	Yes (20 weeks)	no	nd	fetal CCAM or PS or cardiac tumor	none	no	right	40 × 30 × 30	pulmonary artery	not mentioned		pulmonary sequestration
11, Our case	39	3150	M	Yes (20 weeks)	no	cystic mass	ELPS or teratoma intrapericardial mass	150 days	MIS approach + open sternotomy	right	22 × 26 × 27	right pulmonary artery	supra vena cava		pulmonary sequestration

## Data Availability

The raw data supporting the conclusions of this article will be made available by the Authors on request.

## Supplementary Materials

The following supporting information can be downloaded at: https://www.mdpi.com/article/10.3390/jcm15030932/s1, File S1: PRISMA 2020 Checklist. Reference [5] is cited in the supplementary materials.

## Abbreviations

The following abbreviations are used in this manuscript:

PS	Pulmonary sequestration
ELS	Extra-lobar sequestration
CPAM	Congenital pulmonary airway malformations
